# Gamasoidosis (avian mite dermatitis) outbreak in a student dormitory

**DOI:** 10.1590/0037-8682-0228-2024

**Published:** 2024-10-28

**Authors:** Fernando Hiroshi Minagawa, Ettore Rafael Mai Almeida, Rafaela Caroline de Souza, Daniela Carvalho dos Santos, Vidal Haddad, Hélio Amante Miot

**Affiliations:** 1 Universidade Estadual Paulista, Faculdade de Medicina de Botucatu, Departamento de Dermatologia, Botucatu, SP, Brasil.; 2 Instituto de Biociências de Botucatu, Universidade Estadual Paulista, Botucatu, SP, Brasil.

**Dear Editor**

Gamasoidosis is an ectoparasitic dermatosis caused by hematophagous mites belonging to the suborder Mesostigmata. The mite is commonly found in diverse habitats worldwide, such as soil, decomposing organic matter, bird nests, and mammalian burrows. The name of the disease (gammasoidosis) is derived from the order Gamasida (or Mesostigmata). The most prevalent mites responsible for this condition are *Dermanyssus gallinae, D. avium, Ornithonyssus sylviarum*, and *O. bursa*. While it primarily affects animals, such as birds and rodents, human infestations can also occur, resulting in an itchy papular rash that is frequently underrecognized^1^ ^-^ ^6^.

**FIGURE 1:**
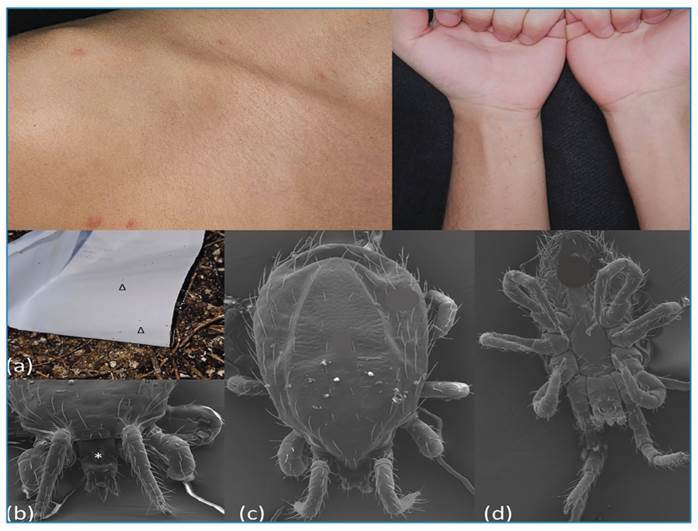
Above: Clinical gamasoidosis. Presentation with multiple erythematous, itching papules on the shoulder, neck, and pectoral regions and erythematous, itching papules on the wrists. Below: **(a)** Empty pigeon nest with a white sheet to demonstrating multiple mites **(Δ)**. **(b)** Anterior view of the mite (*Dermanyssus gallinae*) - Scanning electron microscopic image: 500x. **(c)** Superior view of the mite and entire dorsal shield. **(d)** Inferior view of the mite and genital shield - Scanning electron microscopic image: 224x.

A 25-year-old male university student sought dermatological care due to the onset of disseminated pruritic papules. Four days prior to the consultation, he began experiencing lesions along with a sensation of "bugs crawling on my skin" that intensified when he lay down in bed. Additionally, his girlfriend developed intense itching and erythematous, excoriated papules on her skin two days later. Two colleagues who frequented his room also began experiencing similar itching symptoms (Figure 1).

Subsequently, mites were observed moving on their bodies and bed linen. Despite changing the bed linen, washing clothes, and cleaning the room, the symptoms persisted. Seeking medical care, they were diagnosed with scabies and treated with oral ivermectin; however, the symptoms did not improve.

During the dermatological consultation, mites were discovered on the skin of one of the students, leading to a diagnosis of gamasoidosis. Upon visiting the student dormitory, an empty pigeon nest (*Zenaida auriculata*) was found close to the window and numerous mites were identified and collected manually. They were examined under light microscopy and subjected to scanning electron microscopy for a more detailed examination, which confirmed *D. gallinae* (Figure 1: a,b,c,d). In Brazil*, D. gallinae* can be differentiated from other species of hematophagous parasitic mites on laying hens as they present with an opening in the posterior third of the anal shield, a genitoventral shield that is rounded posteriorly, a dorsal shield that is truncated posteriorly, and a stylet-shaped chelicera. The other species present in Brazil, belonging to the genus *Ornithonyssus* sp., have an opening in the anterior third of the anal shield, genitoventral and dorsal shields that are tapered posteriorly, and a chelicera with two chelae at the distal end^7^.

The students were advised to remove the nest and apply a topical permethrin 5% lotion overnight. Itching was alleviated by oral antihistamines and topical corticosteroids, resulting in complete resolution within 72 hours. 

Gamasoidosis is an important zoonotic disease affecting the poultry industry, particularly in tropical areas. The mites feed on the blood of birds, causing anemia, irritation, feather damage, and in severe cases, death, particularly in young or debilitated birds. Avian mites also act as vectors for various pathogens, including bacteria, viruses, and protozoa, which can potentially lead to the spread of disease among bird populations. Individuals who handle infested birds, travelers, or those who work in environments with high mite populations may develop itchy rashes due to gamasoidosis^3^ ^,^ ^4^.

Urban human gamasoidosis is becoming more frequent because of widespread bird nests, particularly those of pigeons and sparrows, which are commonly found in urban settings on building ledges, under eaves, in air-conditioning units, and in other sheltered locations^1^ ^-^ ^6^.

Human gamasoidosis is characterized by intense and persistent itching, which often worsens at night or following exposure to infested environments. Erythematous papules (1-2 mm in diameter) may develop on various parts of the body depending on the severity of the infestation, with commonly affected areas including the covered regions of the trunk and the nuchal region. As with prurigo simplex, individuals with atopic tendencies often present with more severe manifestations. Although several people visiting the dormitory were affected, skin burrows and lesions were not observed in the genital or interdigital areas. Although mites are occasionally found on skin, they are primarily found in infested environments. Conditions that may present differential diagnoses include scabies, cheyletiellosis (*Cheyletiella* sp.), cholinergic urticaria, and other hypersensitivity reactions to bites from mosquitoes, bedbugs, or fleas^1^ ^-^ ^6^.

As mites require blood for nutrition and reproduction, the treatment of gamasoidosis focuses on eliminating their habitats. *Dermanyssus* sp. can survive in a nest, cracks or crevices of buildings, without food, for up to nine months, in a wide temperature range (5º to 25º C), thus there is potential for reinfestation. The accommodation (dormitory) should be instructed to spray an acaricide (eg., pyrethroid) and/or steam jets in areas close to the removed nest. Topical corticosteroids and oral antihistamines can provide rapid relief from the symptoms^1^ ^,^ ^4^ ^,^ ^5^ ^,^ ^6^.

As gamasoidosis is an emerging zoodermatosis that can occur in any environment, health teams worldwide must be aware of its increasing incidence. It should be considered in cases of acute pruritus with no apparent cause.
